# Long-term outcome after severe traumatic brain injury

**DOI:** 10.1186/cc13660

**Published:** 2014-03-17

**Authors:** M Ciapetti, M Migliaccio, A Cecchi, M Bonizzoli, A Peris

**Affiliations:** 1 Intensive Care Unit, Florence, Italy

## Introduction

Traumatic brain injury is a major public health issue, which results in significant mortality and long-term disability [1,2]. The profound impact of TBI is not only felt by the individuals who suffer the injury but also their caregivers and society as a whole. In this study we observed the long-term outcome of patients with severe traumatic brain injury admitted to our intensive care.

## Methods

This study includes all patients (*n *= 160) with severe head trauma (GCS <9) admitted to the ICU of the emergency department of a tertiary referral center (Careggi Teaching Hospital, Florence, Italy) from 2009 to 2012. All patients will undergo a clinical assessment after 1 year, which is routine post-intensive follow-up. As an objective index of ability to function after injury, the Glasgow Outcome Scale (GOS) will be used. The neurological evaluation to determine the outcome of patients by GOS is performed in two different modes. All eligible patients 1 year after discharge from the ICU are contacted by telephone by a nurse of the intensive care staff and invited to make a visit to the surgery follow-up, where a intensivist evaluates the patient and determines the GOS. For the patients who were still hospitalized at 6 months in rehabilitation departments, GOS assessment is performed by a doctor of the structure and notified by telephone.

## Results

The ICU and hospital mortality was respectively 33.7% (*n *= 54) and 36.9% (*n *= 59). The mortality at 1 year was 44.4% (*n *= 71). The results of the neurological follow-up at 1 year were: GOS 2: 5.6% (*n *= 9), GOS 3: 10% (*n *= 16), GOS 4: 13.1% (*n *= 21), GOS 5: 26.9% (*n *= 43).

## Conclusion

According to the other studies, our data confirm that the severe traumatic brain injury is associated with a high mortality at 1 year. One-half of the survivors have a different level of disability.
